# Nanocracks in nature and industry

**DOI:** 10.1098/rsta.2021.0350

**Published:** 2022-09-19

**Authors:** Kevin Kendall

**Affiliations:** HydrogenUnited.org, 56 Harborne Road, Edgbaston, West Midlands B15 3HE, UK

**Keywords:** nanocracks

This theme issue is a celebration of the paper published half a century ago in *Proceedings of the Royal Society* on ‘Surface energy and the contact of elastic solids' [1]. Every day, a fresh international group publishes research citing this publication about van der Waals attraction, its measurement between solid surfaces and the theoretical interpretation. The objectives of this collection are threefold:

— To describe new experiments illuminating the unexpected adhesion forces experienced between particles, adhesive cracking of nano-materials, the properties of cancer cells, nano-composite materials and so forth
— To consider new theories and consequences of the observed results
— To show how science and engineering must change to consider many particular solutions of the energy conservation equation for nanocracks

My first experience of such fascinating effects came during 1969 at British Railways in Derby where the high adhesion of brown ferric hydroxide nanoparticles was visible everywhere on the rail network (figure 1). Many thousand-tons of iron dust were abraded each year during braking, and the metal particles subsequently corroded to release nanoparticles of about 10 nm diameter that stuck tenaciously to all surfaces, paint and glass alike. These nanoparticles could not be brushed away. They even resisted scraping with a steel blade, leaving oxalic acid dissolution as the preferred cleaning method. By contrast, micro-particles came off fairly easily. Measuring the widely varying adhesive strength of such particulate deposits using pull-off tests showed that there was a significant fundamental problem to be addressed: smaller pull-off probes seemed to register greater adhesion strength. How could this be possible when the van der Waals forces of attraction between the contacting surfaces were constant? It appeared that strength was not definable in the brittle pull-off test, nor in most other brittle-cracking geometries, because force was not proportional to area. This observation goes against the stress theory of cracking first postulated by Galileo in 1638 and also conflicts with the Griffith [2] equation for crack equilibrium that suggests brittle strength should be independent of sample size, but dependent on intrinsic flaw size.

**Figure 1.**
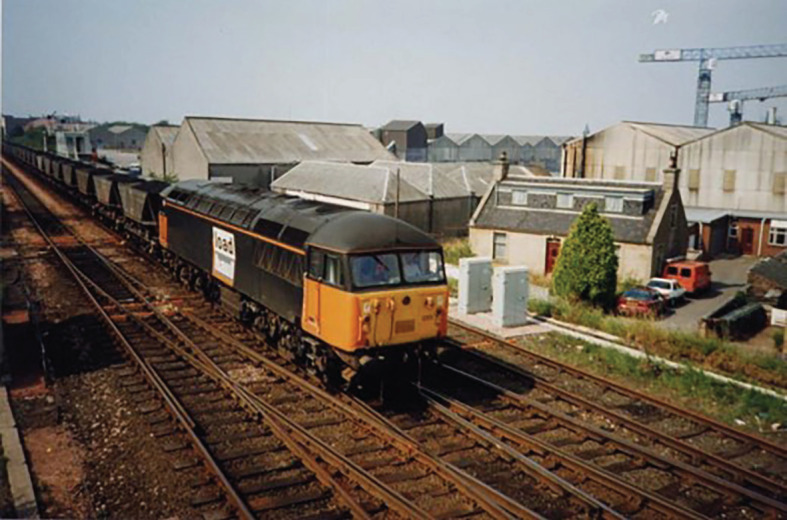
Brown colour of British Railways in the 1960s as cast iron brake-block dust corroded to form oxide nanoparticles 10 nm in diameter, sticking tenaciously to paint and glass surfaces. (Online version in colour.)

There was little doubt that the 10 nm iron hydroxide particles were being pulled by van der Waals attractions into contact with each other while also adhering to the glass/paint surfaces. Since the contact spot size must have been a few nanometres in diameter, fracture of the interface involved nanocracks.

Convincing macroscopic demonstrations of this effect were defined at the Cavendish Laboratory in Cambridge by Alan Roberts and at British Railways by Kevin Kendall using spherical surfaces made by casting hot gelatin solution in a concave glass lens. Roberts had repeated Newton's ring experiments using optically smooth rubber spheres to show a large black contact spot (figure 2) that suggested van der Waals attractive forces were pulling the rubber surfaces together. Newton himself had commented in his 1704 book *Opticks*: ‘The Attractions of Gravity, Magnetism and Electricity … have been observed by vulgar Eyes, and there may be others which reach to so small distances as hitherto escape Observation’. Roberts was the first person to discover these forces acting between macroscopic smooth rubber spheres.

**Figure 2.**
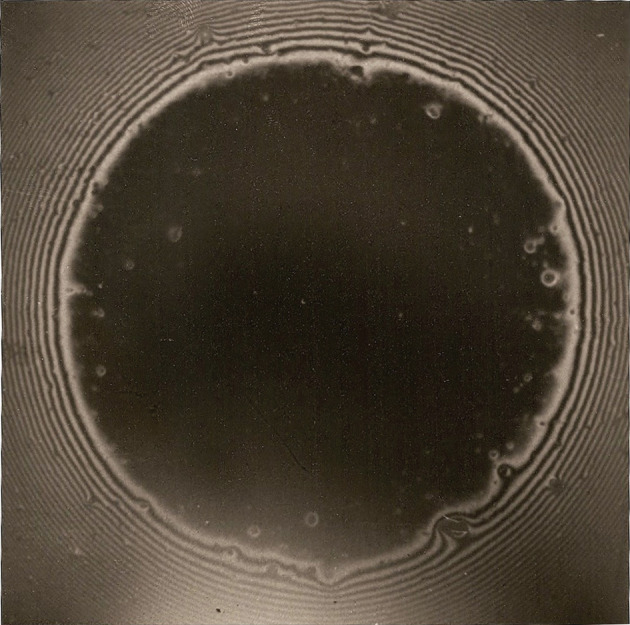
Alan Roberts' micrograph of Newton's ring pattern around the black contact spot between optically smooth rubber spheres. (Online version in colour.)

Ken Johnson, Professor in the Engineering Department at Cambridge, had solved the stress analysis problem for smooth contacting elastic spheres 12 years earlier but had not applied the energy conservation principle of crack equilibrium that Griffith [2] had established a century ago in 1921. Collaboration between Johnson, Kendall and Roberts (JKR) in 1970 changed this and led to the energy balance result [1] giving the equal-sphere pull-off force quite different from Griffith's equation [2]

1.1 $$\text{F}=\frac{3\pi WD}{8},$$

where *W* was the thermodynamic work of adhesion in Jm^−2^ and *D* was each sphere diameter. This caused a shock because we had all been looking for the adhesive strength *σ*, the stress at fracture [3]

1.2 $$\sigma =\frac{F}{A},$$

where *A* was the contact area. Equation (1.1) fitted the experimental results reasonably well. Equation (1.2) did not.

Strength cannot be found in equation (1.1) because the force is proportional to diameter, not area. Also, elastic modulus is absent so *K*_1c_ is not involved. If strength does not exist as a material constant for brittle tests, then the extraordinary powerful adhesion of nanoparticles in figure 1 might be explained. A test on contacting spheres made from solidified gelatin spheres showed that the strength did rise as the diameter was reduced, almost fitting equation 1, as shown in figure 3. The papers in this volume lead us along this new path, which is described more fully in the recent book *Crack Control* [4], showing that brittle strength increases as test samples are made smaller, a fact that has not yet been fully reconciled in Fracture Mechanics textbooks.

**Figure 3.**
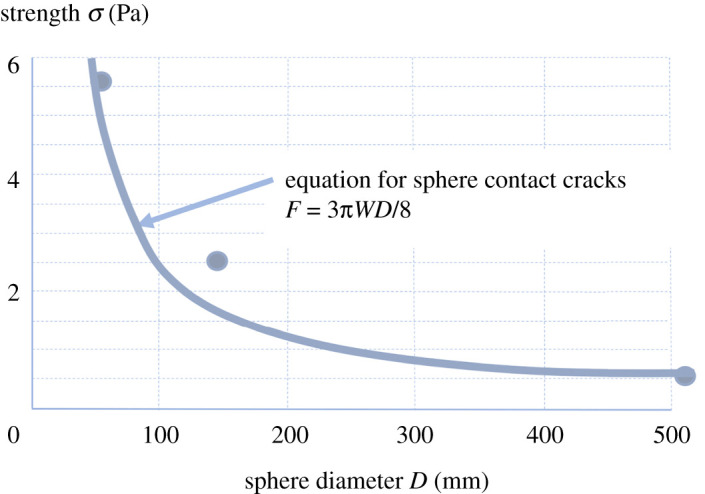
Comparison of JKR equilibrium crack strength equation (1.1) on results for gelatin spherical surfaces of different diameters, showing adhesion strength rises for small sphere diameters as predicted. (Online version in colour.)

The Royal Society has a strong track record in the study of fine cracks, dating back to Hooke's magnificent observations in Micrographia [5] and Newton's celebrated black spot in the ring interference pattern between contacting telescope lenses where ‘two polish'd marbles, … by immediate contact stick together’ to give cracking noises [6,7]. The best Royal Society papers appear in three forms: (i) experimental investigations like the study of rivetted joint strength for iron railway bridges by Fairbairn in 1850 [8]; (ii) theoretical contributions like the Eshelby paper on stress analysis of nano-defects [9]; and (iii) the combination of mathematics and experiment illustrated by the JKR contribution celebrated here [1]. Eshelby's paper is the most cited in the Royal Society *Proceedings A*, while both Fairbairn and Johnson received the Royal Medal of the Society, indicating the perceived social value of crack-engineering that avoids death due to structural collapse. The moral is that theory must go with experiment, as demonstrated in the papers of this present collection on nanocracks.

Griffith's century-old publication in *Phil Trans* [2] provides the background for this special volume on nanocracks, displaying remarkable results of glass tube strength dropping with rising crack length. These results at first fitted his novel thermodynamic theory of crack equilibrium, which happened to contain several significant errors at the time of publication, later corrected such that the theory no longer fitted the experiments [4]. A decade later, Obreimoff [10] presented a much more convincing paper on peeling and splitting of mica, with measurements closely fitting his new theory which completely ignored stress analysis around the crack tip, the dominant feature of Griffith's manuscript, but this was somehow neglected for a century by engineers who did not mention Obreimoff in numerous Fracture Mechanics textbooks, presumably because Obreimoff's equation differs from Griffith's. Obreimoff's cantilever cracking geometry has since been much developed for testing cracks in adhesives and composites [11]. Tabor & Winterton later demonstrated the Obreimoff forces between mica flakes with superb Cavendish laboratory experimentation and theory in the mid-1960s [12].

During that period, Cottrell [13] was a key Cambridge figure in the metals field, and he believed that plastic flow was vital to cracking at the smallest scales and in the most brittle materials. But mica cleavage was omitted (as graphene would be nowadays) because these atomic layers seem to peel apart without any plastic flow. Cottrell also wrote the first paper in Smith's Discussion Meeting collection of 1965 [14], emphasizing cracks in large structures. Following that theme of scaling cracks, one of the most recent collections of fracture papers was written by John Knott and his colleagues [15] in 2014 on different crack sizes across numerous materials, mentioning nanocracks in several papers [16]. They review the history carefully and mention the discovery of atomic defects by G I Taylor [17] and the macroscopic insight into Auerbach's disruptive scaling law by Frank & Lawn [18].

The first paper in this volume [19] describes experiments and theory that add to the original JKR concept by considering very small contacts in which the circular crack has nano-dimensions. This is followed by three papers [20–22] that investigate such JKR nanocracks in various circumstances, including adhesion of biofilm cells, elastic properties of cancer cells and the influence of shear forces that change the behaviour of the JKR cracks as sliding begins to occur. These papers focus on small-diameter contacts through which nanocracks propagate.

In the second group of papers [11,23–25], nanocracks in composite materials are emphasized, starting with carbon fibre-reinforced polymers [11,23], followed by a description [24] of ceramics toughened by graphene nanoparticles. Then, the interesting layered structure of slug-teeth is examined to show that nano-scale toughness can arise in nature [25]. Although macroscopic fracture is observed as the test samples fall apart, it is evident that nanocracks are playing a key role in the initiation of complex damage patterns as the force is increased.

Finally, the last three papers [26–28] consider how the present theories of cracking and sliding need to be adjusted because the original Griffith analysis [2] and its engineering application to Fracture Mechanics require some modifications. In particular, it is important that the original Griffith argument is extended [26] to the case where potential energies of applied forces are added to the Griffith elastic and surface energy terms to define a wider equation for equilibrium. Griffith presumed that the potential term was double the elastic term and negative so could be neglected for fixed grip testing, but in general, the three terms should lead to a quadratic like JKR because (i) surface energy is independent of applied force; (ii) potential energy is typically linear in force; and (iii) elastic energy changes with square of force.

When shear forces dominate, the conservation of energy equation's particular solution can be much more complicated because Schallamach waves of crack opening and healing can be observed [27]. Fracture mechanics has failed to come to terms with such shear phenomena, especially with the size effects that are found in many crack test configurations [28]. For example, lap joints crack at higher stress as the sample size is reduced. Applying the energy criterion of fracture to rubber lap joints shows that the experimental results fit the energy theory. Most striking are the original results by Griffith on the higher tensile strength of finer diameter glass fibres. His results fitted the JKR equation far better than they fitted his own equation. [28] goes on to show that peel cracks, JKR cracks, compression splitting cracks, lap joints and butt joint cracks display this large size effect, which means that nanocracked samples withstand far higher stresses than they should if equation (1.2) was correct. An example of industry relevance is the benefit of small-diameter tubes for hot ceramic fuel cells, which are much less likely to fail under thermal shock when made in smaller diameters. The conclusion must be that ‘strength of a brittle material’ is not a valid idea since the brittle failure force is not generally proportional to cross-section area, though the Griffith equation suggests otherwise. This fits the opening remarks in this introduction, where nanoparticles adhere thousands of times stronger than stress theory predicts. We conclude that nanocracks require higher stress to propagate than macro-cracks and so must be viewed differently in nature and in industry. Fracture Mechanics has failed to emphasize this point, although a recent book does connect Fracture and Size Effect for concrete failure [29], while [4] is 100% based on smaller samples displaying higher fracture stresses.

My thanks are due to the Royal Society, the organizing committee for this volume, Dr Bohm, Prof Alford, Prof Clegg and Prof Kinloch, and to all authors in this special nanocrack collection

## Data Availability

This article has no additional data.
